# Keep calm and carry on: moral panic, predatory publishers, peer review, and the emperor's new clothes

**DOI:** 10.5195/jmla.2022.1441

**Published:** 2022-04-01

**Authors:** Frank Houghton

**Affiliations:** 1 frank.houghton@lit.ie, Director of Social Sciences ConneXions, Technological University of the Shannon, Limerick, Ireland

**Keywords:** predatory publishing, peer review, academic quality, scientific misconduct, Elsevier, academic journals

## Abstract

The moral panic over the impact of so-called predatory publishers continues unabated. It is important, however, to resist the urge to simply join in this crusade without pausing to examine the assumptions upon which such concerns are based. It is often assumed that established journals are almost sacrosanct, and that their quality, secured by peer review, is established. It is also routinely presumed that such journals are immune to the lure of easy money in return for publication. Rather than looking at the deficits that may be apparent in the practices and products of predatory publishers, this commentary invites you to explore the weaknesses that have been exposed in traditional academic journals but are seldom discussed in the context of predatory publishing. The inherent message for health and medical services staff, researchers, academics, and students is, as always, to critically evaluate all sources of information, whatever their provenance.

## INTRODUCTION

A review of recent publications on the issue of predatory publishing and predatory journals reveals an ongoing focus on this topic [1–7]. However, it is important that assessments of the potential impacts of such predatory journals are not overplayed, and it is likely true to say that there has been a certain degree of hysteria over the would-be threat from such journals [8].

First, it must be made clear that the world of commercial academic publishing is a highly profitable oligopoly [8, 9, 10]. It must immediately be asked, therefore: Whose interests are served by the moral panic over predatory publishers? The Oxford English Dictionary describes a moral panic as

a mass movement based on the false or exaggerated perception that some cultural behaviour or group of people is dangerously deviant and poses a threat to society's values and interests. Moral panics are generally fuelled by media coverage [11].

**Figure 1:**
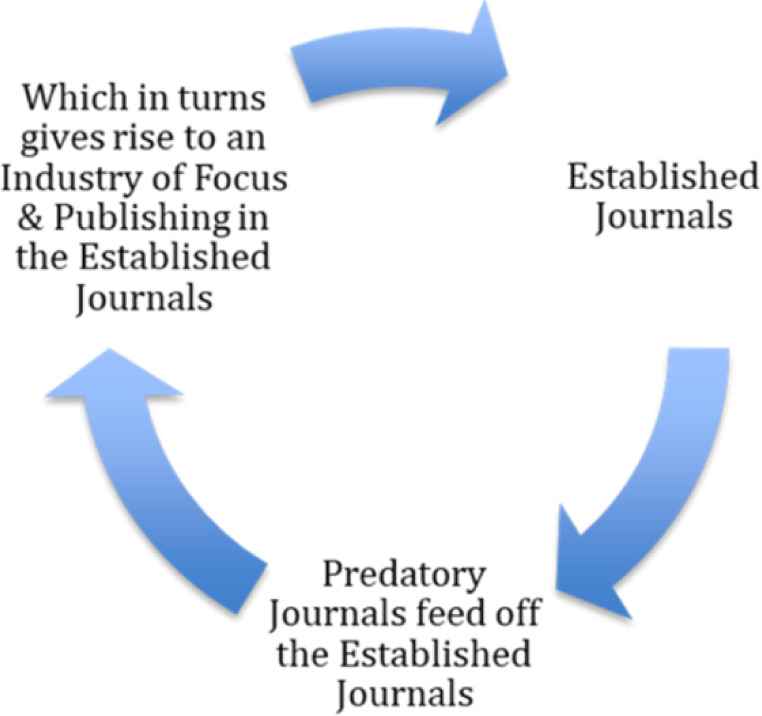
The symbiotic nature of the established & predatory publishing nexus

Predatory publishing is often portrayed as threatening the very basis of science and, by extension, our way of life. It must be acknowledged that there is an intense academic focus on predatory publishing within the academic literature. This may almost now be described as an industry in its own right. It appears that we now have a symbiotic relationship in which predatory publishers exist due to mainstream journals, and the mainstream journals themselves now use the existence of such predatory journals as a focus for further publishing. This stark parallel may be seen in Figure 1.

Despite the attention paid to the threat of predatory journals, examinations of citations from them indicate that the actual impact of such publications appears limited [12–14]. As such, the reality may be that their impact is similarly marginal. Bell suggests that predatory journals are not really a threat but instead should be treated as parody [8]. Instead, perhaps solicitations from such journals should be met “with amusement (and annoyance) rather than alarm” [8]. An ongoing concern is the potential conflation of concerns about, and attacks on, predatory publishing and open access (OA) publishing [15].

It must also be acknowledged that some of the venom that has been targeted at predatory journals and some of the general language used in the field to discuss the issue may have racist overtones [16–18].

For example, as well as the routine use of the term “blacklist” to denote a negative list [18], Jeffrey Beall, the founding and leading proponent of a virtual crusade against predatory publishing, has written such posts as:

Hyderabad, India is one of the most corrupt cities on earth, I think. It is home to countless predatory open-access publishers … and new, open-access publishing companies and brands are being created there every day … The tacit rule of thumb of Hyderabad-based businesses is: Use the internet to generate revenue any way you can. There are numerous internet-based businesses in this over-crowded city [18].

It is hard to forget or forgive this blanket labeling of an entire city and its population as corrupt, and his use of the term over-crowded appears only to stoke racist and xenophobic imagery and ideology.

Much of the high-profile criticism that has been leveled at the predatory publishing sector is based on a series of sting operations where authors submitted patently low-quality articles for publication [19–26]. However, it is essential to note that it is not only so-called predatory journals that are routinely fooled. For example, in the infamous Bohannon sting operation [21], in which a series of fake and fatally flawed scientific papers were sent to hundreds of pay-to-publish journals, as well as being accepted by widely acknowledged predatory publishers, the papers were also accepted by journals published by “reputable” publishers such as Sage, Elsevier, and Wolters Kluwer. Equally, the well-known Sokal hoax, in which a physicist published a nonsensical paper (“Transgressing the Boundaries: Towards a Transformative Hermeneutics of Quantum Gravity”) in a cultural studies journal, also involved what was widely considered a leading journal in the field [25, 26].

It is therefore crucial to critically evaluate the mainstream academic literature that is generally held in such high regard vis-à-vis predatory publishers. Perhaps the most infamous case that may require a sober reappraisal and leveling of esteem in the field of publishing is evidenced by Elsevier, a global leader in academic publishing. Elsevier Australia was exposed publicly for receiving payments from a pharmaceutical company for publishing six fake journals (*Australasian Journal of General Practice, Australasian Journal of Neurology, Australasian Journal of Cardiology, Australasian Journal of Clinical Pharmacy, Australasian Journal of Cardiovascular Medicine, Australasian Journal of Bone & Joint Medicine*), all of which advocated the efficacy of the products of the sponsoring pharmaceutical company [27, 28]. As Goldacre notes:

Elsevier Australia went the whole hog, giving Merck an entire publication which resembled an academic journal, although in fact it only contained reprinted articles, or summaries, of other articles. In issue 2, for example, nine of the 29 articles concerned Vioxx, and a dozen of the remainder were about another Merck drug, Fosamax. All of these articles presented positive conclusions. Some were bizarre: such as a review article containing just two references [27].

Although this is perhaps an extreme example, many will agree that the cornerstone of quality control in academic publishing is peer review. Peer review has long been established as the gold standard in academic circles. However, even a cursory examination of the literature on this topic reveals this process as a mirage akin to the emperor's new clothes.

Drummond Rennie, deputy editor of the *Journal of the American Medical Association,* has stated that “if peer review was a drug it would never be allowed onto the market” [29]. In a damning exposé of peer review, Rennie is quoted:

There seems to be no study too fragmented, no hypothesis too trivial, no literature citation too biased or too egotistical, no design too warped, no methodology too bungled, no presentation of results too inaccurate, no conclusion too trifling or too unjustified, and no grammar and syntax too offensive for a paper to end up in print [29].

There is significant evidence that peer review routinely fails to detect significant errors, even in reputable journals [29–37]. Undoubtedly one of the most robust examinations of inadequacies in the academic peer review process can be seen in an alarming examination published by the *Journal of the Royal Society of Medicine* [38]. This study involved an “insider” research approach in which three articles were deliberately weakened after having been accepted for publication. The articles in question were amended to include nine major errors and five minor errors. These critically weakened articles were then sent out for peer review to over 600 *BMJ* peer reviewers, with between 418 and 522 reviewers taking part in examining each of the three papers. Disconcertingly, out of the nine major errors introduced, the average number of errors spotted by the peer reviewers ranged from just 2.58 (SD=1.9) to 3.05 (SD=1.8). Of the five minor errors introduced, the average number noted by the reviewers was just 0.85 (SD=0.8) to 1.09 (SD=0.8). It must be acknowledged that some reviewers recommended rejection before identifying all of the errors, and that some reviewers may not have been familiar with the methodology of randomized control trials (RCTs) but might have performed better reviews in assessing other methodologies. Some caution in interpretation may also be required in relation to this paper, as it was restricted to UK reviewers and hence may not be generalizable. However, and perhaps more disturbingly, this study sought to determine if training in peer review would improve the quality and found that there was no significant improvement [38]. Despite such damning evidence, Smith quite correctly states that “when something is peer reviewed it is in some sense blessed” [39]. It must be acknowledged that there is strong evidence to suggest that when reviewers are asked to review a paper, levels of agreement on whether it should be published or not is little better than would be anticipated by chance alone [29, 40, 41].

The so-called predatory publishing field is routinely attacked on the basis of poor quality, the implication being that quality is far higher among established journals operating the traditional publishing models. However, having now raised significant queries over the quality and impact of peer review on traditional journals, it is now opportune to explore a number of other weaknesses in such journals that are also seldom, if ever, linked to the debate around predatory publishers. A host of other critical deficits could be explored within mainstream academic journal articles ranging from publication bias [42–47] to academic plagiarism [48, 49] and routinely poor statistical methods [50–53]. However, for reasons of brevity, this commentary will focus on just two such issues as exemplars to demonstrate the weaknesses that appear inherent in the world of academic publishing that are seldom discussed vis-à-vis predatory publishing: errata and the issue of scientific misconduct and retractions.

## ERRATA

Starting with errata, it should be noted that even highly prestigious “traditional” journals also routinely include errors in their publications [54, 55]. A recent examination by Hauptman et al. [56] revealed that almost a quarter (24%) of articles examined in such journals included at least one significant error, which “materially altered data interpretation” [57]. A subsequent examination of five top-ranking journals in the medical field (*New England Journal of Medicine, Annals of Internal Medicine, British Medical Journal, Journal of the American Medical Association*, and *The Lancet*) over a twelve-month period identified 314 articles with one or more published errata, an average of 1.3 per issue [57]. Even when errata are published, it has been noted that these can take a significant length of time to appear [57]. In examining this issue, it is perhaps more alarming to note the work of Molckovsky et al., who found that:

33% of oncologists do not read errata, and 45% have read only the abstract when referencing an article. Although 59% of oncologists have noticed errors in cancer publications, only 13% reported the error [58].

Having established the inadequate treatment of errata by publishers of mainstream academic publications, the next section will explore another aspect of publishing that is also seldom mentioned in the predatory publishing debate, that of scientific misconduct and subsequent retractions. This will help to demonstrate the moral panic over predatory publishers is overstated, ignoring the weaknesses of traditional academic publishing.

## SCIENTIFIC MISCONDUCT & RETRACTIONS

It must be acknowledged that although some editors may, rather naively, underplay the impact of scientific misconduct in the academic literature, it is a growing issue [59]. It is hard to know if this is indicative of fraud levels rising, or of awareness and policing of the problem developing. However, either way, it is clear that there has been a significant focus on this issue in recent years [60, 61]. A *British Medical Journal* survey revealed that 13% of respondents reported being aware of fraudulent data manipulation [62], with another similar study putting this figure at 14% [63]. It should also be noted that although fraud-based retractions are not a new phenomenon [64], there is widespread agreement that retractions based on fraud have increased dramatically in recent years [65, 66]. Notably, a review of significant examples of such retractions is published annually in *The Scientist* [67–70].

## DEALING WITH ERRORS AND FALSIFICATION

Some of the long-term issues noted above in relation to errata, covering what may be termed honest mistakes, are also cause for concern in relation to retractions of fraudulent articles [71–73]. For example, Elia et al. discuss how few articles are fully and properly retracted following exposure [74]. Interestingly, one of the reasons reported by these authors for this deficiency includes publishers not printing the retractions as requested. These authors suggest that “retractions appear to be unpopular with both editors and institutions since they may shed doubt on the integrity of science, and on the expertise of the editorial team” [74]. Once fraudulent or erroneous findings have been published, evidence suggests that despite exposure as false, such work may continue to be cited positively, sometimes for decades [71, 75–79]. Retraction Watch have what they term a Leader Board of such issues [80]. At present, the leader in this list is a 2013 article in the *New England Journal of Medicine* that was retracted in 2018 [81]. The article has a total of 2,623 Web of Science citations, 712 of which have occurred after the retraction of the paper. Interestingly, the infamous MMR *Lancet* article, which takes second place, was published in 1998 and has been cited in Web of Science more since its retraction in 2010 than before it (820 versus 643) [82]. It must be acknowledged that some citations may be to refute an article or to discuss aspects other than the results. However, retracted articles continue to be routinely cited; the vast majority are routinely building on the work of an article, and this remains a highly problematic issue [75–78]. It should be noted that although such errors and misconduct are often seen to irretrievably damn new online publishers and journals, no such sector-wide approach is taken to these issues when they appear in more mainstream traditional journals.

## CONCLUSION

This paper is not designed to be an argument in support of a race to the bottom in terms of quality. However, it is designed to reduce the “blessedness” referred to by Smith in relation to established academic journals [39] and to acknowledge that a more nuanced response may be appropriate [8]. The moral panic evident in the academic literature heralding the doom of academic publishing because of the rise of predatory journals is misplaced. Traditional journals routinely demonstrate the poor practices that are held up as damning in predatory publishers. The sensationalism and crusading zeal launched against suspected predatory publishers has had its unintended casualties. Some indication of the collateral damage on OA journals can be seen in Emery and Levine-Clair's article provocatively titled “Our lives as predatory publishers” [15]. Loose attacks on predatory publishers may indirectly threaten OA journals generally, and it is important to remember the vested interests of the established academic publishing oligopoly in fostering such concerns [9]. Considerable literature has emerged designed to help authors identify fraudulent predatory publishers [83, 84]. This will be useful to would-be authors. However, although the provenance of a journal may be important, more crucial is teaching critical evaluation skills. An excellent journal article can easily be published in a predatory journal. Similarly, a weak and dangerous article may be published in a reputable journal [82]. In the tradition of Feyerabend, students, academics, and health service staff should question everything and critically interrogate all information that they are presented with and be prepared to speak out and challenge it [85].
